# Kunafa knife and play dough is an efficient and cheap simulator to teach diagnostic Point-of-Care Ultrasound (POCUS)

**DOI:** 10.1186/s13017-018-0220-3

**Published:** 2019-01-08

**Authors:** Fikri M. Abu-Zidan, Arif Alper Cevik

**Affiliations:** 1 Department of Surgery, College of Medicine and Health Sciences, UAE University, Al-Ain, 17666 United Arab Emirates; 2 Department of Internal Medicine, College of Medicine and Health Sciences, UAE University, Al-Ain, 17666 United Arab Emirates

**Keywords:** Ultrasound, Simulation, Point-of-care, Basic physics, Fanning, Teaching, Learning

## Abstract

**Background:**

Point-of-Care Ultrasound (POCUS) is a useful diagnostic tool. Nevertheless, it needs proper training to reach its required level of competency. Educators who work in low-income countries find it difficult to purchase expensive training computer-based simulators. We aim in this communication to describe the methods to build up and use an efficient, simple, and cheap simulator which can be used for teaching POCUS globally.

**Methods:**

It took our group 2 years to develop the simulator to its current form. The required material for the simulator includes a Kunafa knife, a carton gift box and its cover and colored play dough. The Kunafa knife with its blade is an excellent simulator for the small print convex array probe (3–5 MHz) and its ultrasound sections. It is useful to teach two important principles. *First*, the three basic hand movements used to control the ultrasound probe (fanning, tilting, and shifting). *Second*, the thin blade of the knife (1 mm thick) simulates the shape of the two-dimensional ultrasound images. The play dough is used to simulate different organs to be cut in different directions like the aorta and inferior vena cava.

**Results:**

The simulator was used to teach 88 fifth year medical students during the period of November 2017 to November 2018 at the College of Medicine and Health Sciences, UAE University. The simulator was valid, simple, portable, and sustainable. The students greatly enjoyed its use. The cost of the simulator is less than 10 US dollars.

**Conclusions:**

Surgical educators who work in low-income countries are encouraged to develop their educational tools that are tailored to their own needs. Our simulator can help our colleagues who want to teach POCUS and cannot purchase expensive mannequins and computer-based simulators.

## Introduction

Point-of-Care Ultrasound (POCUS) is currently used through all stages of surgical care including resuscitation, preoperative diagnosis, surgery, and intensive care [1, 2]. It is a **P**hysiological **O**n spot clinical decision tool which is used as an extension of the **C**linical examination in a **U**nique and **S**afe way for managing critically ill patients [3–5]. Understanding its principles and limitations is the first step towards mastering this diagnostic tool and avoiding its pitfalls. Nevertheless, POCUS is operator dependable and needs proper training to reach its required level of competency. That is one of the hallmarks for a successful use of POCUS in managing critically ill patients [6–8]. Therefore, POCUS training given for undergraduate medical students is recommended [9].

Globally, there are huge differences in economical status, educational, technical, and health care facilities between different countries which will definitely impact training in acute care surgery and critical care medicine. The annual gross national income (GNI) per capita is less than 1000 US dollars for low-income countries and more than 12,000 US dollars for high-income countries [10]. These differences lay heavy burdens on educators who work in settings having few resources. Knowledge and understanding of the local facilities and their needs will help in developing educational methods that are tailored to the income of these countries.

The World Society of Emergency Surgery advocates developing clinical guidelines that are accepted globally including low-income countries [11]. Furthermore, it is developing surgical programs that aim at helping low- and middle-income countries. We have recently developed an efficient, simple, and cheap simulator that can be used for teaching POCUS globally. This stemmed from more than 30 years of intense experience in both clinical and educational POCUS activities. We aim in this communication to describe how to build up and use this simulator so as to help our colleagues in low- and middle-income countries who want to teach POCUS and do not have enough resources to purchase expensive mannequins and computer-based simulators.

## Methods

### Participants

This method has been taught to 88 undergraduate fifth year medical students during their junior surgical clerkship at the College of Medicine and Health Sciences, UAE University by the first author (FAZ) during the period of November 2017 to November 2018. There were 20 groups of 3–5 students each, 70 were females (80%) and 18 were males (20%).

### Teaching structure

Each teaching session took two and half hours in which the first hour was used to teach the basic physics of ultrasound including tissue impedance, modifying the ultrasound waves, and sonographic artifacts. A standard simple illustrated book chapter was used as a reference for these topics for medical students [12]. The simulator was used to demonstrate two important concepts (1) hand movements and (2) three-dimensional mental mapping. The students were then taken to 90-min bedside clinical teaching on patients demonstrating the learned skills.

### The Kunafa knife

**Fig. 1:**
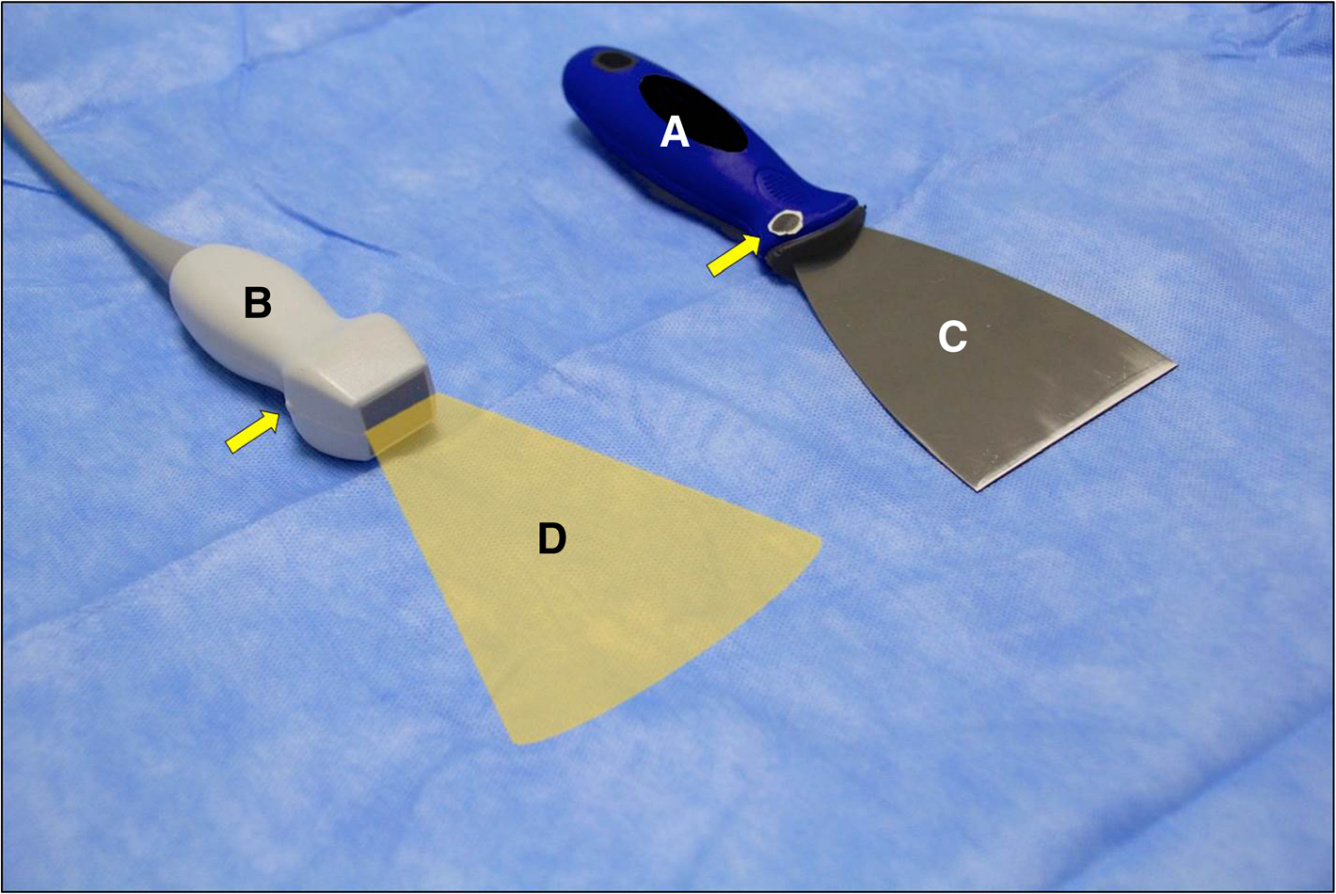
The Kunafa knife is an excellent simulator for the small print convex array probe (3–5 MHz) and its sections. The handle of the knife (**a**) simulates the probe (**b**) while the blade (**c**) simulates the cross sections (**d**).The yellow arrow points towards the probe pointer

**Fig. 2:**
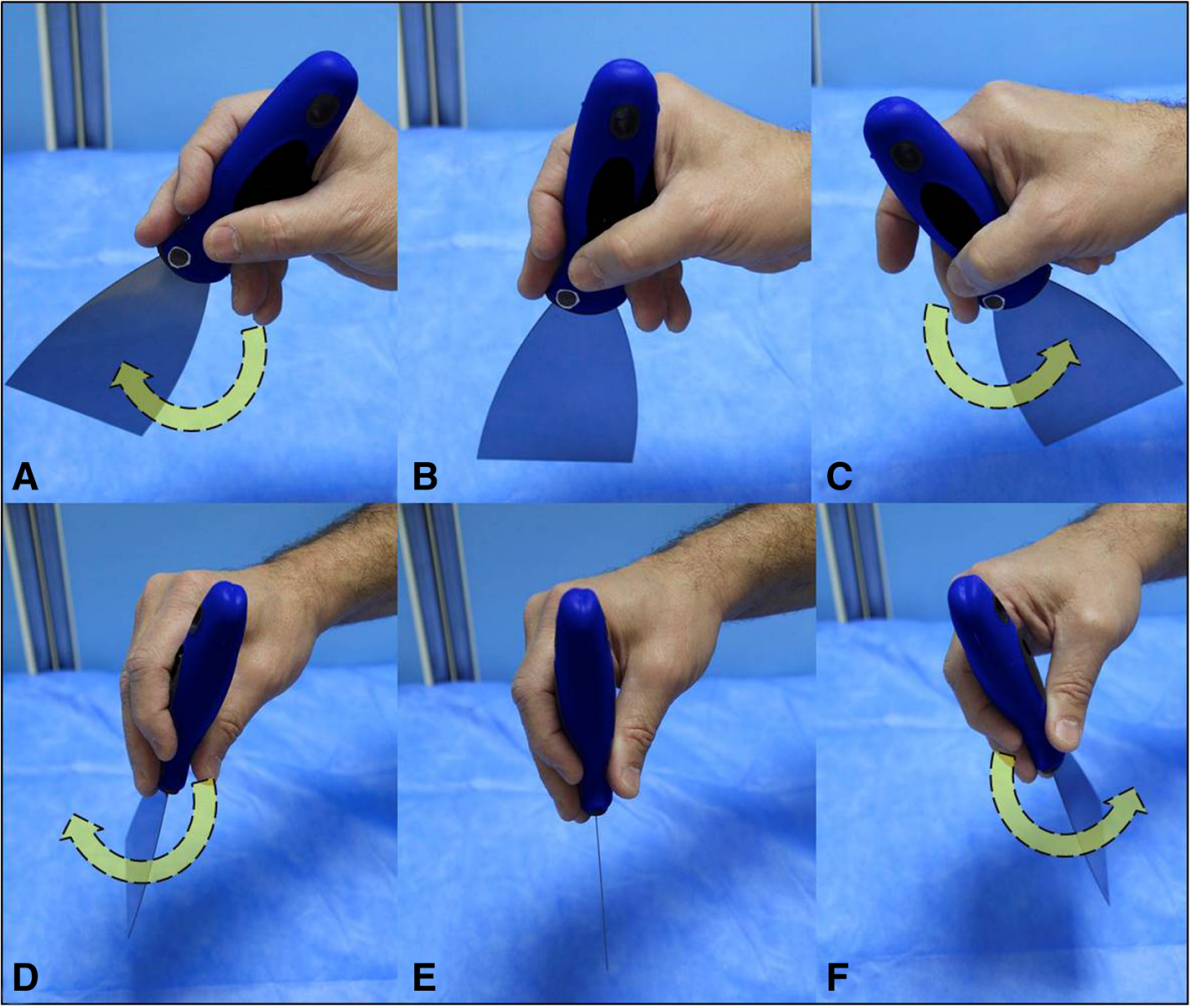
The Kunafa knife is useful to understand the longitudinal fanning of both the sagittal **b** mode sections (**a**–**c**, curved yellow arrows) and transverse **b** mode sections (**d**–**f**, curved yellow arrows)

The Kunafa knife is an excellent simulator for the small print convex array probe (3–5 MHz) and its sections. The handle of the knife simulates the probe while the blade simulates the B mode section **(**Fig. 1**)**. The Kunafa knife is useful to demonstrate two important principles. *First*, the three basic hand movements used to control the ultrasound probe which includes (a) the fanning **(**Fig. 2**)**, (b) the tilting (changing the angles of the probe on the surface from sagittal (0 °) to transverse (− 90 °) without fanning it) and (c) the shifting (moving the probe completely from its place to another). The marker of the probe should always be directed up (to the head) or to the right side of the patient because acute care physicians may scan different body cavities at the same time [12]. *Second*, the two-dimensional (2D) brightness (B) ultrasound image is just as thin as the blade of the Kunafa knife (1 mm thick) and resembles its shape (Fig. 1). Reading an ultrasound image is similar to reading a single page of a story book. To understand the whole ultrasound story you have to fan the ultrasound probe so that you can read all pages of the story.

### The POCUS simulator

**Fig. 3:**
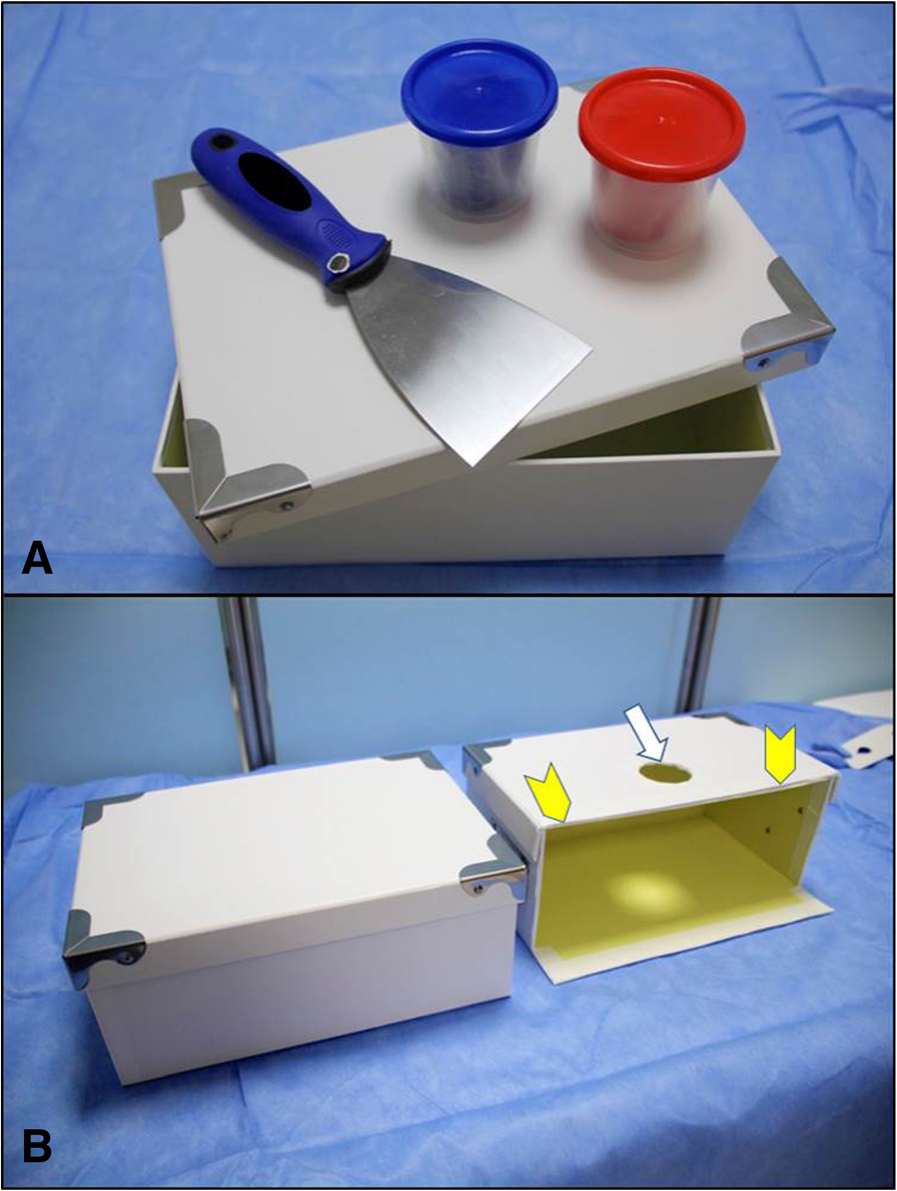
The required material for the simulator includes a Kunafa knife, a carton gift box and its cover and colored play dough (**a**). A circle having a diameter of 5 cm is cut at the center of the cover (white arrow). Both the box and cover were cut 4 cm away from the side (arrow heads) while sparing the floor of the box (**b**)

**Fig. 4:**
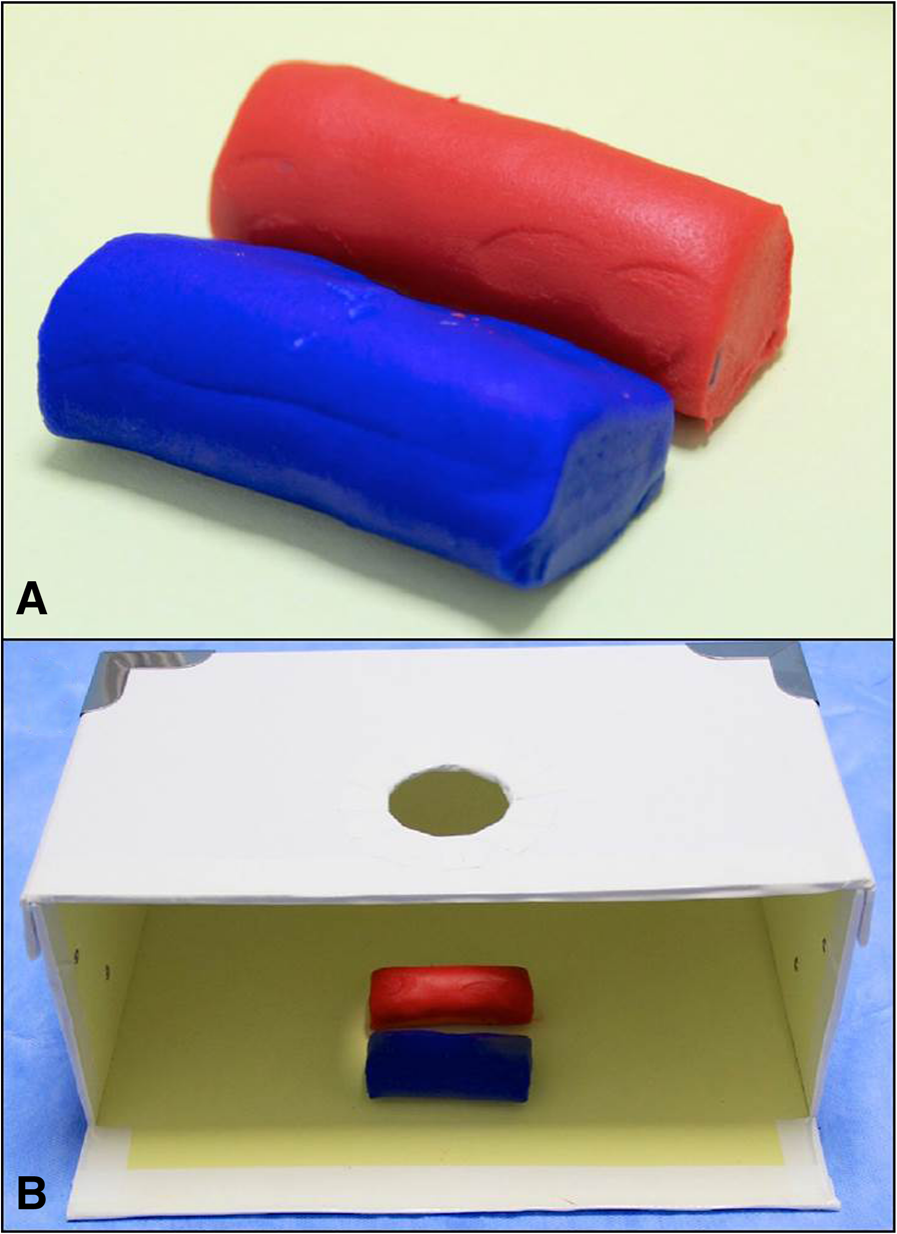
The play dough is used to simulate different structures (**a**) like the aorta (red) and the inferior vena cava (blue). The gift box simulates the abdominal cavity (**b**)

**Fig. 5:**
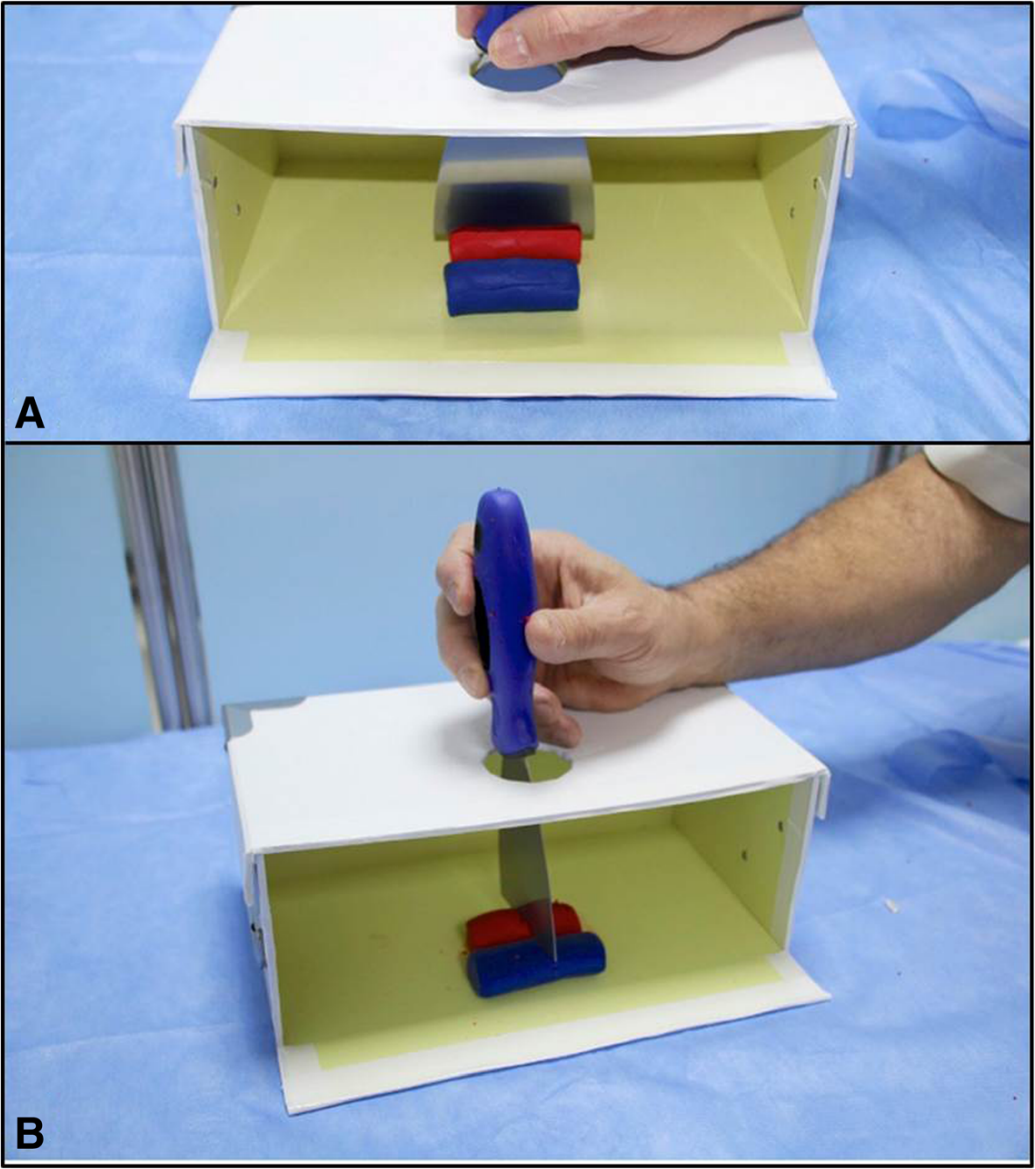
The handle of the Kunafa knife is passed through the hole in the lid of the box and then the students can observe the outcome of the longitudinal sections (**a**) and transverse sections (**b**) of different structures, the aorta (red), and inferior vena cava (blue) as shown in this example

The required material for the simulator includes a Kunafa knife, a carton gift box and its cover (28 × 11 × 19 cm) and colored play dough (Fig. 3). A circle having a diameter of 5 cm is cut at the center of the cover. Both the box and cover are then cut 4 cm away from the side (arrow heads) while sparing the floor of the box. The gift box resembles the abdomen, the Kunafa knife resembles the ultrasound probe and its ultrasound section, while the play dough is used to simulate different organs to be cut, like the aorta (red color) and IVC (blue color) **(**Figs. 4 and 5**)**.

### Using the POCUS simulator

**Fig. 6:**
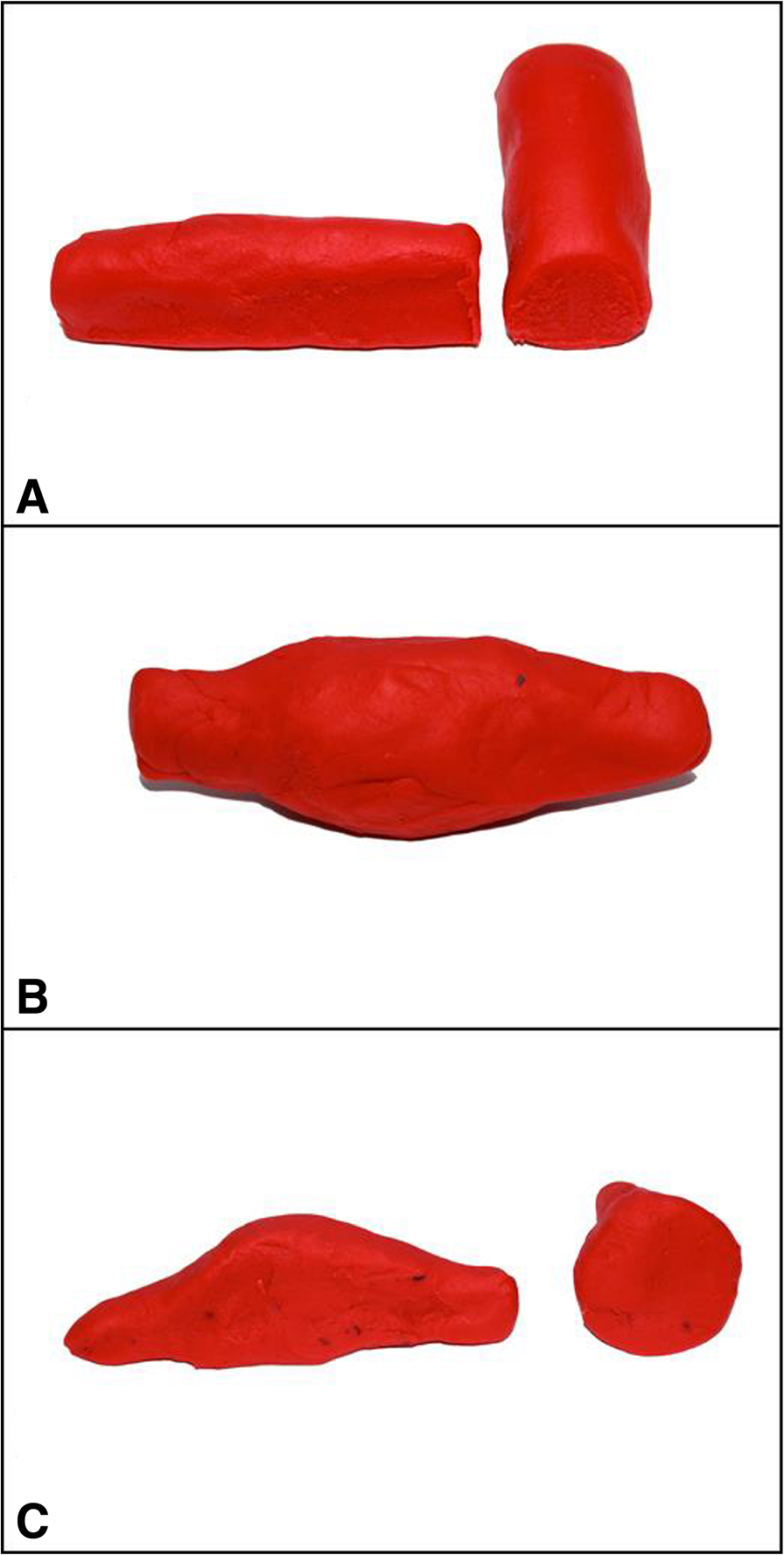
An image of the play dough demonstrating the outcome of a longitudinal and transverse cross sections of an aorta (**a**), an aortic aneurysm (**b**), and a longitudinal and transverse cross sections of an aortic anurysm (**c**)

**Fig. 7:**
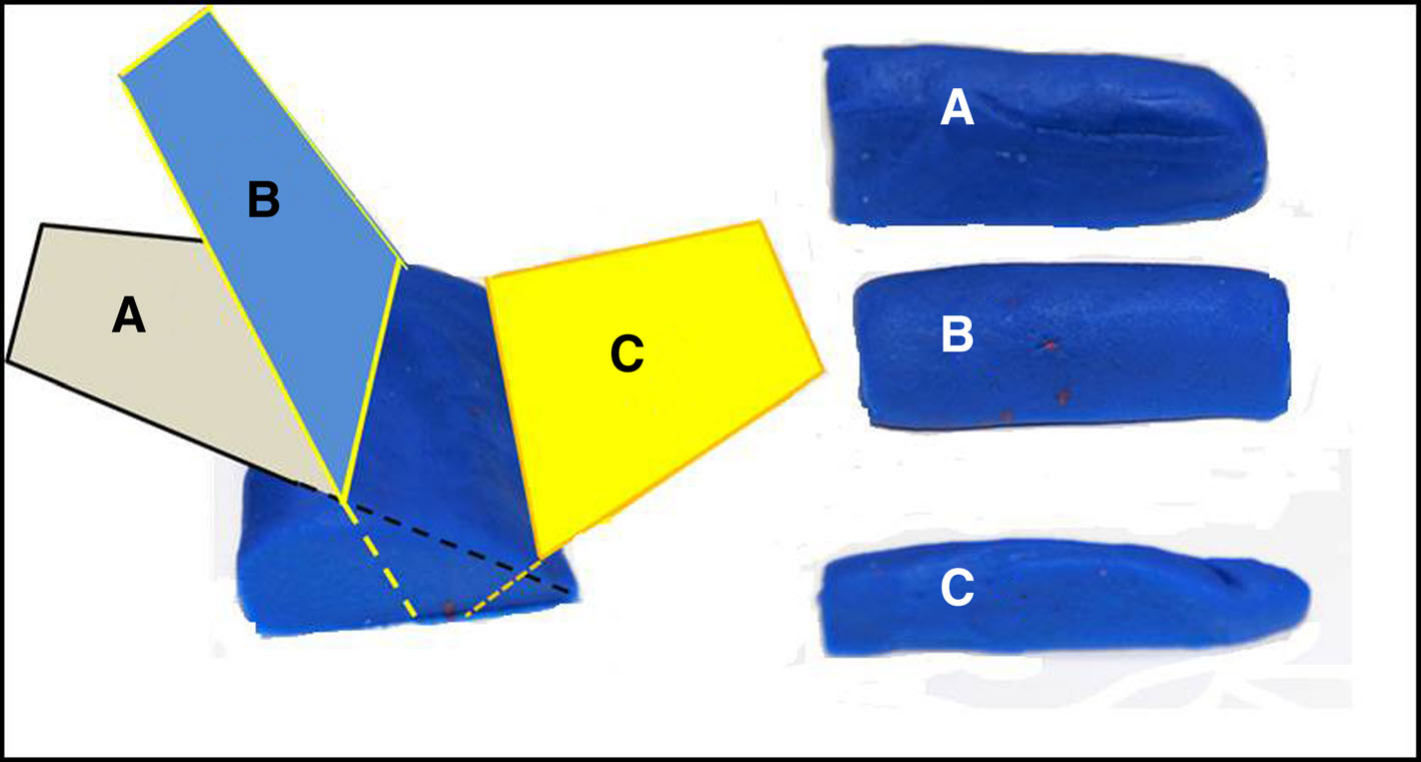
An image of the play dough demonstrating the outcome of longitudnal sections of IVC at different planes (**a**–**c**). The two common pitfalls in measuring the IVC diameter is cutting it obliquely (**a**) which gives a false bigger diameter, or peripherally (**c**) which gives a false smaller diameter

The students initially used the Kunafa knife to perform three essential movements (fanning, shifting, and tilting). They are asked to slow down when they fan so as not to miss findings. Furthermore, cross sections of the play dough were demonstrated to show how the image would look like when cutting the play dough through different planes. Scanning the aortic aneurysm and measuring inferior vena cava diameter are excellent examples to demonstrate the value of this model (Figs. 6 and 7).

### Aortic aneurysm simulation

The principle of scanning the aorta by transverse sections from the epigastrium to the bifurcation to diagnose an aortic aneurysm were demonstrated by cutting the aortic aneurysm model transversely at different levels so that the student can appreciate the change from normal diameter to a dilated aneurysm and finally to a normal diameter and then the bifurcation of the aorta **(**Fig. 6**)**.

### Measuring the IVC diameter simulation

The IVC shape is like a tree leaf (not rounded) where the narrower part is at the medial side (Fig. 7). The IVC play dough is cut longitudinally at different planes to demonstrate the effect on the measured IVC diameter (A–C). The two common pitfalls in measuring the IVC diameter is cutting it obliquely (A) which gives a false bigger diameter, or peripherally (C) which gives a false smaller diameter. The students are then taught that using the transverse section for measurement of the IVC is the best method because the smallest achieved section of the IVC is the proper section which was cut vertically. This allows direct vertical measurement of the vein at its center using the moving (M) mode.

### Direct feedback of the students

Following that, the students were directly asked about their perceptions and opinions on this educational method, whether they found it useful in clarifying the technical aspects of both the manual movements and the three-dimensional mapping of ultrasound, and whether they enjoyed it.

### Bedside training

The students were then directly taken to selected real trauma patients on whom the extended focused assessment sonography for trauma (EFAST) was performed using the 8 Ps and I sonographic windows. These included the following views in sequence: *p*erihepatic, right *p*leural recess, *p*erisplenic, left *p*leura recess, *p*elvic (both sagittal and transvers views), *p*ericardiac (subcostal view), right *p*neumothorax and left *p*neumothorax (at the second mid-clavicular line) and finally the *i*nferior vena cava diameter measurement through an anterior intercostal approach [2, 13]. The B mode and M mode were used to measure the IVC diameter both in the longitudinal and transverse cross sections. The findings are explained by the sections which have just been achieved by the play dough.

## Results

The students enjoyed the exercise and were highly supportive of its use. They thought that this method has simplified ultrasound for them, that this is the first time they could understand how ultrasound works, and that they were excited to learn ultrasound in their future clinical career. Some have expressed that they are planning to buy their own portable machine to master this diagnostic tool.

We, as educators, quickly found that this model has different important characters (by surface validity) which were strongly supported by the student’s direct comments. These are

- *Validity*: The tool reached exactly what it was meant and planned for. It was clear that the students understood the meaning of fanning and imagined that ultrasound is just a thin section (1 mm thick). The students could quickly and easily imagine the cut sections which were taken depending on the marker of the probe and its position.
- *Simplicity*: This simulator can be easily kept in a small tool box and carried in a bag with the instructor to the hospital. It does not need electricity and can be assembled within less than 2 min.
- *Enjoyment*: The students usually get excited, open their eyes, and start laughing when they see a Kunafa knife (which is a common dessert in our community) and wonder how it would be used for teaching ultrasound. They get stimulated, quickly get engaged, and try to understand what the relationship between the Kunafa knife and ultrasound is. Using play dough is fun for them as most properly it reminds them of their childhood. So learning becomes play and fun instead of being rigid science.
- *Cost effectiveness*: The detailed costs of the components of the simulator are shown in Table 1. The total cost of the simulator is less than 10 US dollars.
- *Portability*: The simulator is light, takes small space, does not need electrical battery or connection, and can be easily carried to different educational places.
- Sustainability: The play dough set was used in all 20 training sessions without getting dry.

**Table 1:** The material and its cost which were used to construct the Point-of-Care Ultrasound (POCUS) simulator

Item	Cost (US dollars)
Kunafa knife	1.9 $
Three colored dough (100 g)	3.27 $
White gift box (28 × 11 × 19 cm)	2.73 $
White masking tape	1.33 $
Tax (5%)	0.46 $
Total	9.69 $

## Discussion

We have developed a cheap useful simulator using the Kunafa knife and play dough to teach POCUS for undergraduate medical students. We think that this portable simulator is a simple, valid, and cost-effective educational tool that may help colleagues in low-income countries in teaching POCUS.

We have recently reported our prospective global study on the management of acute appendicitis (MAGIC study) which included 4271 consecutive patients. There was significant variation in the radiological workup of patients depending on the GNI per capita [14]. CT scan was done in less than 8% in the low-middle income countries compared with 38% in high-income countries. In contrast, ultrasound was done in more than 70% in the low-middle income countries compared with 60% in the high-income countries. The recent guidelines of the World Society of Emergency Surgery for the management of acute left colonic diverticulitis support the use of ultrasound as an initial diagnostic tool [15]. The major two limitations for using ultrasound globally are availability and training.

During 2017, the annual GNI per capita for different countries ranged globally between 290 dollars and 82,650 US dollars [16]. This has major impact on health care and educational resources. There have been great examples of innovative successful cheap clinical solutions in low-income countries like using a sterilized mosquito mesh in hernia repair instead of the expensive commercial mesh [17]; and using a sterile soup ladle, kitchen funnel, sterile gauze, and a glass bottle to collect patient’s own blood for auto-transfusion [18]. A similar approach should be adopted to develop educational methods for teaching POCUS in low- and middle-income countries. These should be innovative and stemming from deep understanding of the needs and problems encountered.

To develop this model, there was a need for in depth knowledge of the basic physics of ultrasound combined with considerable practical experience on teaching POCUS. The first author (FAZ) had been involved in developing complex animal models for teaching POCUS besides using human models in different POCUS courses in different countries for almost two decades [6–8]. The simulator was built up based on the basic principles of ultrasound imaging in a cheap and practical way. The basic mode which is commonly used to perform POCUS is the two-dimensional (2D) brightness (B) mode [19, 20]. Abdominal ultrasound examination for detecting free intra-peritoneal fluid, pleural fluid and peri-cardiac fluid is performed using the small print convex array probe (3–5 MHz) [19]. This probe gives wider and deeper views [12, 21] and its sections are very similar to the Kunafa knife blade. The ultrasound operator should have a clear three-dimensional mental anatomical map when performing the ultrasound study.

In 2009, we were astonished to find how a knife and an apple were very useful in teaching echocardiography views when used as a simulator in a low-middle income country [22]. In comparison and 10 years later, we have used the Kunafa knife in the current simulator. Kunafa (or Kanafeh) is a common traditional dessert of the Middle East which is made from thin noodle-like pastry, soaked with sugar-based syrup, and layered with cheese [23]. It is cut using the Kunafa knife into square pieces [24]. Observing the similarity between the Kunafa knife and the small print convex array probe gave us the idea of using it as a simulator to teach POCUS. This idea stemmed from familiarity with the ultrasound probe and its function. The simple idea went into different stages of development, trial, and refinement during 2 years to reach the current reported form.

In a systematic review, we have recently compared different teaching focused assessment sonography for trauma (FAST) models including computer simulators [25]. Computer simulators were used in 15% of the reported studies. They produce patients’ three-dimensional ultrasound images that are reconstructed in real time when scanning a mannequin. This makes POCUS teaching and assessment more objective. The mentor can select normal or abnormal images as he/she wishes [26]. Those who are trained on simulators can properly identify pathological ultrasound images similar to those who are trained on real patients. This reduces the need for clinical patients, allows for safe training [26, 27], and affords a standardized learning experience [26–28]. Furthermore, they can be used for mastery training in which a predetermined level is designed. The learners continue practicing till they reach that level with little involvement of mentors [29, 30]. Nevertheless, this training method has certain limitations. First, the fine manual practical skills which are needed in real ultrasound scanning, like avoiding the rib shadow artifacts by fanning or shifting, are difficult to teach by using a computer simulator. Second, the qualities of ultrasound images of intra-abdominal organs that vary with human or animal body built are difficult to mimic [8]. Third, they may be expensive and may not be affordable by many institutions. Fourth, the critical decision-making using POCUS in sick patients is best taught bedside in the real life.

### Limitations

It is important to highlight that this study has certain limitations. First, it may be argued that the reported method is just a personal experience and not an educational outcome study. Personal educational experience is a well-accepted subjective educational research method. Educationist when merged in their educational experience sense what works and what does not work by comparing their recent experience with their previous ones. We may be biased towards our new teaching model. Nevertheless, we think and hope that our simulator will support teaching POCUS globally especially in low- and middle-income countries. This opinion is stemming from more than 30 years deep interest and personal experience in this area.

Second, our simulator will not replace or produce ultrasound images. We think that the cost of expensive computer simulators should be invested in buying portable ultrasound machines that can be used directly on bedside for clinical care, education, and research at the same time. Ultrasound is very safe, and students can directly practice ultrasound on real patients following understanding its principles. That is different from training on invasive procedures like laparoscopy which has risk on patients. We have been using this approach for teaching POCUS, and we find it useful, feasible, and practical. This gives us the advantage of applying POCUS to solve clinical problems and show students how this works in real life as an on spot critical decision tool.

## Conclusions

Surgical and acute care educators who work in low- and middle-income countries are encouraged to develop their own educational tools that are tailored to their own needs. We have developed a simulator for teaching POCUS which is cheap, valid, simple, portable, enjoyable, and sustainable. The cost of the simulator is less than 10 US dollars**.** We hope that our simulator will support teaching POCUS globally especially in low- and middle-income countries.

## Abbreviations

2D: Two dimensional
B mode: Brightness mode
EFAST: Extended focused assessment sonography for trauma
FAST: Focused assessment sonography for trauma
IVC: Inferior vena cava
M mode: Moving mode
POCUS: Point-of-Care Ultrasound
